# Learning dialogs orchestrated with BookRoll: effects on engagement and learning in an undergraduate physics course

**DOI:** 10.1186/s41039-022-00203-0

**Published:** 2022-07-22

**Authors:** Vijayanandhini Kannan, Jayakrishnan M. Warriem, Rwitajit Majumdar, Hiroaki Ogata

**Affiliations:** 1 Department of Physics, GITAM School of Sciences, GITAM (Deemed to Be University), Rudraram, Hyderabad, 502 325 India; 2 NPTEL, Indian Institute of Technology Madras, Chennai, India; 3 Academic Center for Computing and Media Studies, Kyoto University, Kyoto, Japan

**Keywords:** Learning dialogs, Learner-centric MOOCs (LCM Model), Learning and evidence analytics framework (LEAF), Technology-enhanced and evidence-based education and learning (TEEL) platform, Active learning, Emergency remote teaching, Physics education

## Abstract

With COVID-19 pandemic forcing academic institutions to shift to emergency remote teaching (ERT), teachers worldwide are attempting several strategies to engage their learners. Even though existing research in online learning suggests that effectiveness of the online session is more dependent on pedagogical design rather than technology feature, teachers may still focus on the intricacies of the technology. In this paper, we present the evolution of an active learning pedagogy, supported by technology (eBook reader—BookRoll, Analytics Dashboard—LAViEW), for an undergraduate physics classroom across a semester that was affected by the lockdown due to pandemic. The technology-enhanced pedagogy evolved in three phases—technology used in “Content Focus” mode, technology used in “Problem Focus” mode and technology used in “Learning Dialogue Focus” mode. The entire activities were designed and implemented within the technology-enhanced and evidence-based education and learning (TEEL) ecosystem, which supported integration of learning technologies with analytics system. Comparison of the student’s learning logs indicated that there was a sustained engagement in the learning activities conducted during the blended (before lockdown) and online mode (during lockdown). We had conducted one-way ANOVA to compare the post-test scores for each teaching phase and found statistically significant differences in the latter phases. A preliminary qualitative analysis of the learner artifacts generated as memos in BookRoll during each phase revealed that students were posing conceptual clarifications during the latter phases. These were also having greater alignment with the session agenda and showed construction of new knowledge based on the seed knowledge provided during the instructor–learner interaction sessions. The study provides key insights into how reflection and practice by both learner and teacher improves the acceptance of technology-enabled pedagogy.

## Introduction

The COVID-19 pandemic had forced institutions worldwide to transition into online teaching mode. However, the abruptness of this transition has resulted in “Emergency Remote Teaching (ERT)” strategies (Hodges et al., 2020) being used unlike a planned online remote teaching. Counter to the existing research on online teaching and learning, where need for promotion of learner self-reflection, self-regulation and self-monitoring is highlighted for positive learning outcomes (Means et al., 2009), the ERT strategies are predominantly focused on mimicking standard classroom experience. This would mean that faculty teaching in ERT settings needs to start focusing more on the pedagogical aspects rather than technology skills for ensuring success of the online course experience (Garrison et al., 2010; Shieh et al., 2008). This is a challenging objective as the known barriers of attitude and skill deficit for online teaching–learning practices among teachers (Keengwe & Kidd, 2010) will become a major roadblock in this transition phase.

In the current paper, we describe the evolution of an online learning strategy in an undergraduate Physics course for engineering students in India. The instructor of the course had been utilizing the Technology-enhanced Evidence-based Education and Learning (TEEL) platform (Ogata et al., 2018) that integrates an LMS (MOODLE), eBook Reader (BookRoll) and Learning Analytics Dashboard (LAViEW). She has been using this for more than a year and had shown earlier that the adaption of active learning strategies in flipped learning methods using the TEEL platform had been effective to better engage the students and enhance learning (Kannan & Gouripeddi, 2019). The authors had also reported about the better student engagement during the COVID-19 lockdown (Kannan et al., 2020a) by adopting a technology-enhanced pedagogy facilitated by the TEEL platform. We extend this work by tracing the evolution of this technology-enhanced pedagogic strategy over a semester of instruction affected by COVID-19 and explore the significance of reflective practice (Schön, 1987) both by the learner and by the teacher. The technology-enhanced pedagogy evolved in three phases—“Lecture Focus” (L) mode, “Problem Focus” (P) mode and “Learning Dialogue (LeD) Focus” (also called L + P) mode. The study investigates the engagement as per logged data as well as the transitions across different performance groups of the students for different activity design and discusses the implication for teaching and designing the activities in an online setting.

The following research questions were investigated:

- RQ1: Is there any difference in engagement between low and high scoring students during the lecture focus, problem-solving focus and LeD focus phases?
- RQ2: What is the variation in the learning performance of low and high scoring students in assessments conducted at the end of each of the three phases—lecture focus, problem focus and LeD focus?

In the paper, we have interchangeably used LeD and L + P terminologies as the LeD focus is a combination of lecture (L) and problem (P) focus phases.

The paper is organized as follows: The “Foundations” section presents the key understanding of the existing literature relevant to improvement in engagement in online learning; “Pedagogical foundations” section analyzes the proposed pedagogical and technological foundation of the current work; “Research methods” section explains the methodology adopted for analyzing the data and answering the research question; “Results” section discusses the answers to the research questions along with qualitative analysis of artifacts generated and instructor reflection; in the “Discussion and conclusion” section, we elaborate the impact of results obtained in our study.

## Foundations

### Improving engagement and learning in an online setting against the backdrop of COVID-19

An analysis of relevant literature related to online learning shows that the use of innovative online tools when combined with the careful design of learning activities boosted the self-motivation and efficacy of online learners (Alqurashi, 2019; Jenna & Gillett-Swan, 2017). For instance, Whipp and Lorentz (2009) suggests that teacher–students’ interaction and engagement can be enhanced by asking conceptual questions frequently and thereby providing timely and concise feedback to those seeking help. These questions trigger the process of reflection among students to provide a much higher level of critical response and aid in the process of knowledge construction (Gerber et al., 2005). Yet another study found that tools or features promoting self-reflection among students lead to better learning outcomes in chemistry, language learning, physics and math problem-solving (Means et al., 2009). Jensen and Scharff (2014) highlights the need for incorporating multiple opportunities for practice and feedback so as to develop critical reading skills among learners through eBook readers. Lin et al. (2017) have reported on how to appropriately shape the instructional design of online lecture videos to effectively engage learners.

Study by Dixson (2010) points to the need for developing multiple communication channels between learners and instructors in an online environment to improve student engagement. This is in line with the ideas of social and teaching presence (Garrison et al., 2010) in the community of inquiry framework or the characteristics of E-learning environments as mentioned in the E-learning Engagement Design (ELED) framework (Czerkawski & Lyman, 2016). These channels will help instructors understand their students better and promote strategies that are pertinent to the students’ need (Chen & Jang, 2010).

To summarize, there have been sufficient pointers to effective teaching–learning practices in an online setting which recommended sustained engagement and motivation, of both the instructor and the learner, with the technology as a necessary prerequisite for making learning more effective. (Chiu et al., 2021; Losier et al., 2001).

The sudden shift to online learning during pandemic did not provide this opportunity for either the instructor or the learner to develop this sustained engagement with technology. Online learning practices have not been predominantly favorite among teachers (Pomerantz & Brooks, 2017) as this requires them to shift from a teacher-centered pedagogy to a student-centered pedagogy (Schmidt et al., 2016). Oliver et al. (2002) have identified that creating online learning instruction material takes more time and requires deeper content knowledge and creativity from the teachers. Therefore, it becomes overwhelming for teachers to stay focused on multiple teaching tasks mediated with technology, such as delivering the content, follow-up activities, focus on tools, assess and provide feedback of learning (Kebritchi et al., 2017). The literature also provides substantial evidence that the educational research practitioners and novice instructors lack adequate skills to exploit the technological affordances for a particular teaching–learning context (Conole & Dyke, 2004). Studies done during the initial period of lockdown further show that the perceived student learning and satisfaction is influenced by instructors' facilitation and knowledge of the online learning environment (Baber, 2020). Vera et al. (2020) have conducted a detailed student’s survey related to technology and its accessibility while shifting to online learning during lockdown. Their findings suggested that students also faced several discomforts due to the lack of familiarity with technology. The study showed that the majority of respondents expressed difficulties to effectively navigate between multiple technologies like learning management or collaboration tools (Canvas, Zoom, Slack, etc.) and instructor’s or textbook Web sites during the synchronous online sessions. Many studies have recently pointed out that student’s experienced learning fatigue, Internet challenges and access to devices during the ERT (Wilcox & Michael, 2020). Further, the students were reported to be unclear of the expectations around the technology being used and faced troubles to find an adequate digital replacement for having a better instructor–student interaction.

In the current study, the technology platform—technology-enhanced evidence-based education and learning (TEEL), integrates a learning management system (MOODLE), an eBook reader (BookRoll) and learning dashboard (LAViEW) which will be detailed in the upcoming sections. The instructor had been using the BookRoll and MOODLE technologies available in this platform for flipped classroom implementation over the past year and had observed better engagement from her learners for an active learning strategy implemented through the system (Kannan & Gouripeddi, 2019; Kannan et al., 2020a). However, the scenario of lockdown with a new batch of students was indeed a new challenge for the instructor as well.

### Motivation

The instructor has been using the BookRoll and MOODLE features of the TEEL platform for more than a year, prior to lockdown, for facilitating blended teaching–learning practice. The lockdown imposed as a result of the pandemic required the instructor to innovate on her practices and quickly adapt the TEEL platform features for the fully online pedagogy in the middle of the semester. TEEL platform supports the integration of technologies with learning analytics systems to assist the seamless orchestration of our active learning pedagogy in blended and online learning settings. The current set of students in the course had roughly been exposed to the features of the TEEL platform for not more than 2 months and the instructor herself was exploring the analytics dashboard (LAViEW) for the first time. In spite of these limitations, the instructor came up with a strategy called learning dialog (LeD) orchestrated with BookRoll to mitigate the concerns related to ERT. This strategy was developed based on her face-to-face experience with the same batch of students. Thus, the main motivation of this study is to trace how the teacher was able to develop a new pedagogic strategy in the face of a significant challenge and understand how this benefitted her students in the process of teaching–learning.

### Pedagogical foundations of current work: LeD in the LCM model

**Fig. 1:**
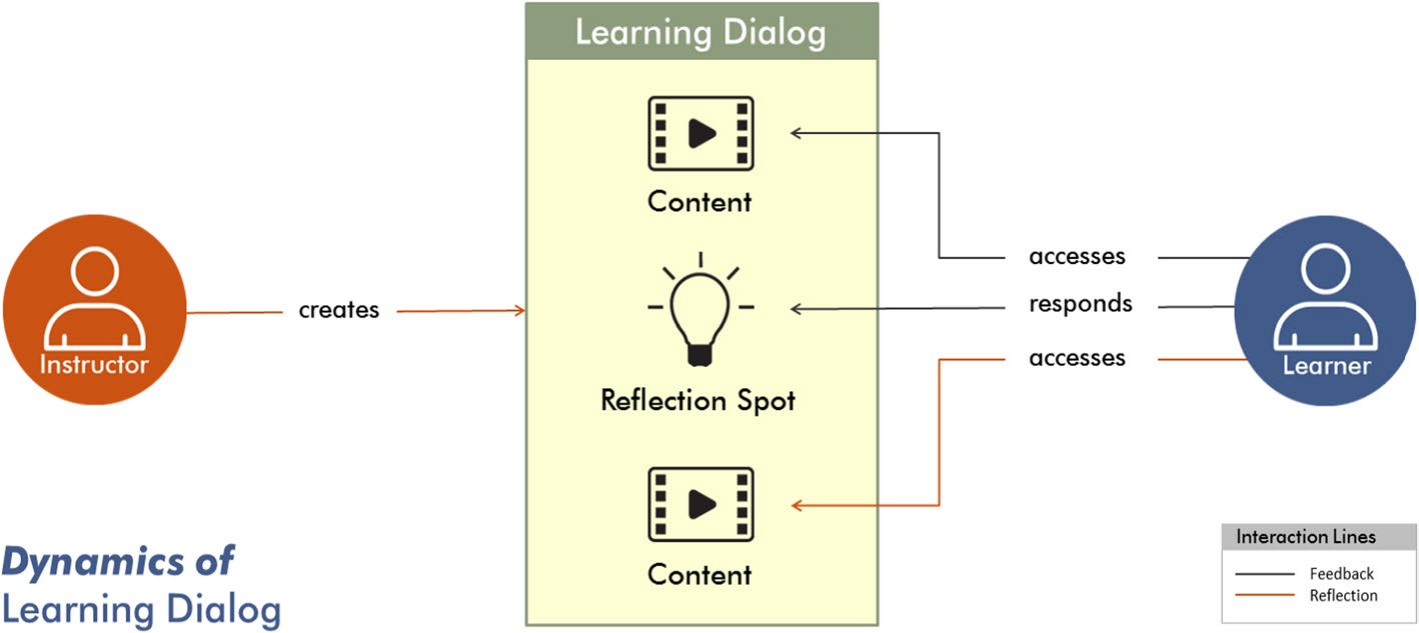
Dynamics of learning dialog in the learner-centric MOOC (LCM) model (Murthy et al., 2018)

The learner-centric MOOCs (LCM) offer a prescriptive model consisting of a set of guidelines, activity formats and actions for MOOC creators (Murthy et al., 2018; Shah et al., 2022). The model emphasizes interactive activities rather than traditional information transfer and subscribes to the larger philosophy of learner-centricity. The Learning Dialogues (LeDs), in the LCM model, promote concept acquisition through learner interaction. As seen from Fig. 1, a key design feature of an LeD is the “Reflection Spot,” a place within the content access where the instructor permits the learner to express prior conceptions, perform micropractice or reflect. The content can be in any of the multimedia formats—video, text, image, etc. The reflection spots are implemented as question prompts and are followed by an explanation, feedback or summary by the instructor to close the learning loop. There can be more than one reflection spot in the content, depending on the length and complexity of the topic being discussed.

### Technology-enhanced evidence-based education and learning platform

**Fig. 2:**
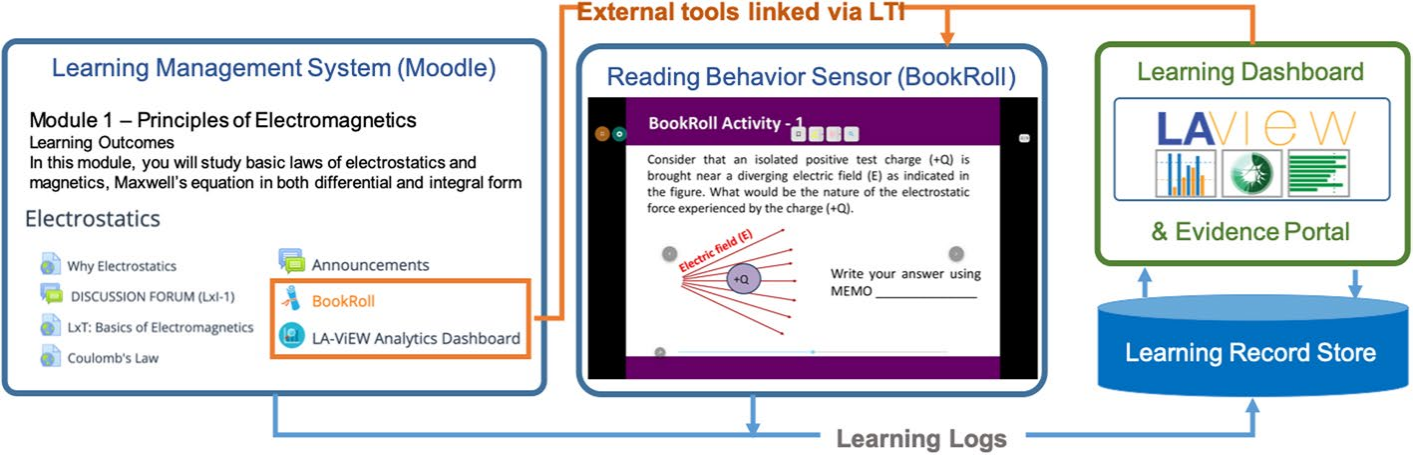
Components of TEEL framework in this study (Kannan et al., 2020a)

We orchestrated our course on the TEEL platform from January till April, 2020. Figure 2 shows the four major components of TEEL. The learning behavior sensor captures the learner's and teacher's interaction data during the session. It offers an LMS (MOODLE) that integrates other e-learning tools through LTI standards—eBook Reader (BookRoll) and learning analytics dashboard (LAViEW)(Ogata et al., 2015; Shimada et al., 2018) . BookRoll allows student to read digital contents such as lecture slides or materials that are shared by the instructor. It has a feature like red or yellow markers to highlight some parts of the text that are important or difficult to understand. Additionally, students can add memos to remember important points, annotate doubts or comments. They can bookmark pages to access them easily while reviewing the content. These actions are recorded and then can be viewed by the instructor to understand the reading habits of students in the learning analytics dashboard LAViEW (Majumdar et al., 2019). LAViEW contains various panels of visualized indicators for monitoring and plays a central role to assist and identify problems in the teaching–learning scenario based on analysis of the visualized indicators. Both teachers and students can access these learning tools. Thus, the TEEL platform integrates the features of the eReader, LMS and Dashboard within a single service so that teachers can seamlessly move across the technology.

### Evolution of the strategy: LeD with BookRoll

As indicated in the earlier sections, even though the instructor was familiar with the TEEL platform for more than a year, the current semester saw a new batch of students taking up her course. Based on the learnings from earlier work (Kannan et al., 2020a, 2020b) , the instructor had initially facilitated demonstration sessions of the technology inside the classroom and then provided the learners with focused activities in the BookRoll for eliciting learner artifacts in the form of BookRoll memos. The LAViEW dashboard provided the instructor with a view of these artifacts which then subsequently helped them in devising feedback to the learners. As seen from Table 1, the pedagogic design evolved over three phases. The first two designs happened in blended mode (Pre-COVID), while the third happened in completely online mode (during lockdown).

**Table 1:** Evolution of the strategies in the TEEL Platform over the semester

Mode	Pedagogic strategy	Content elements based on LeD			Objective of the strategy	Technology–Pedagogy Integration
		Content	Reflection Spot	Feedback		
Blended mode (pre-COVID lockdown)	Lecture focus (L)	Face-to-face lecture (synchronous)	BookRoll Activity of Clarification Spot—Students generate memo related to their conceptual queries (Asynchronous)	Face-to-face Feedback sessions to clarify common misconceptions/queries	To support student reflection	The Reflection Spot is operationalized in an asynchronous manner, where students note their queries as memos in BookRoll. The LAViEW tool helps the teacher to aggregate this so that a feedback session can be planned for clarifying the queries
	Problem-solving focus (P)	Face-to-face lecture (synchronous)	Bookroll Activity of Reflection Spot—Students do problem-solving through BookRoll Memos (Asynchronous)	Face-to-face Feedback sessions to cover common solution approaches	To help student to micropractice	The Reflection Spot focuses on micropractice and hence require students to note their solutions as memos. The LAViEW tool helps the teacher to aggregate this and identify the common solution approaches taken by them. Subsequently a feedback session is conducted for discussing these approaches
Online mode (during COVID lockdown)	LeD focus (L + P)	Online lecture	Two type of BookRoll Activities: 1. Doubts posted during clarification Spot 2. Micropractice of solutions at Reflection Spots	Feedback is provided during the same session for both queries and problem solutions	To engage students in reflection and also allow micropractice	The reflection spot integrates both students reflection and micropractice through BookRoll Memos. The teacher is able to look at the aggregate response in LAViEW and provide feedback in the same session

### Lecture focus phase (L phase)

**Fig. 3:**
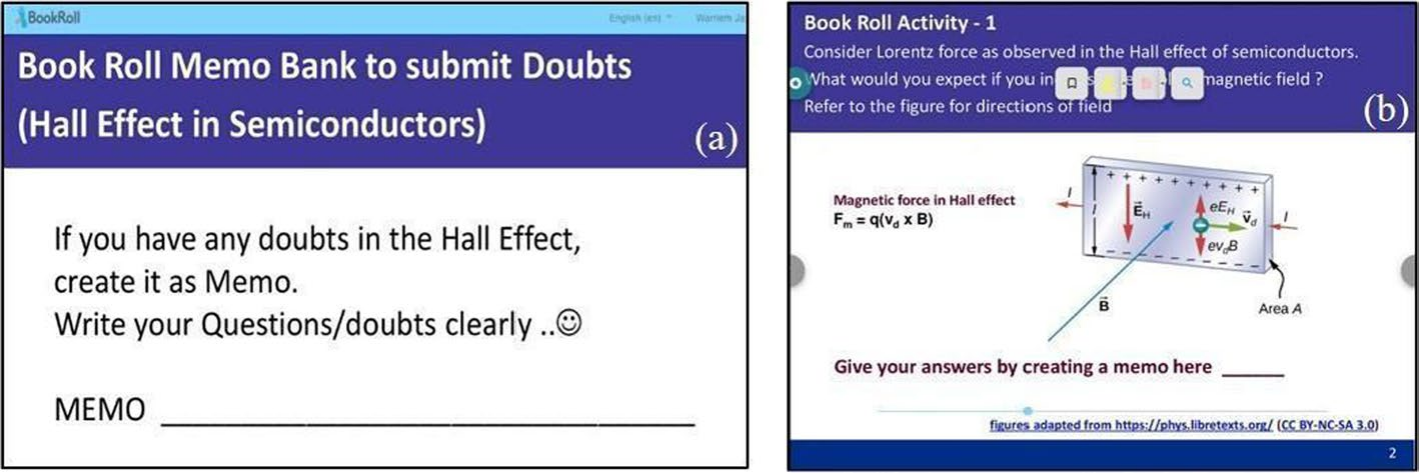
Examples of **a** clarification spot and **b** reflection spot activity created in BookRoll

**Fig. 4:**
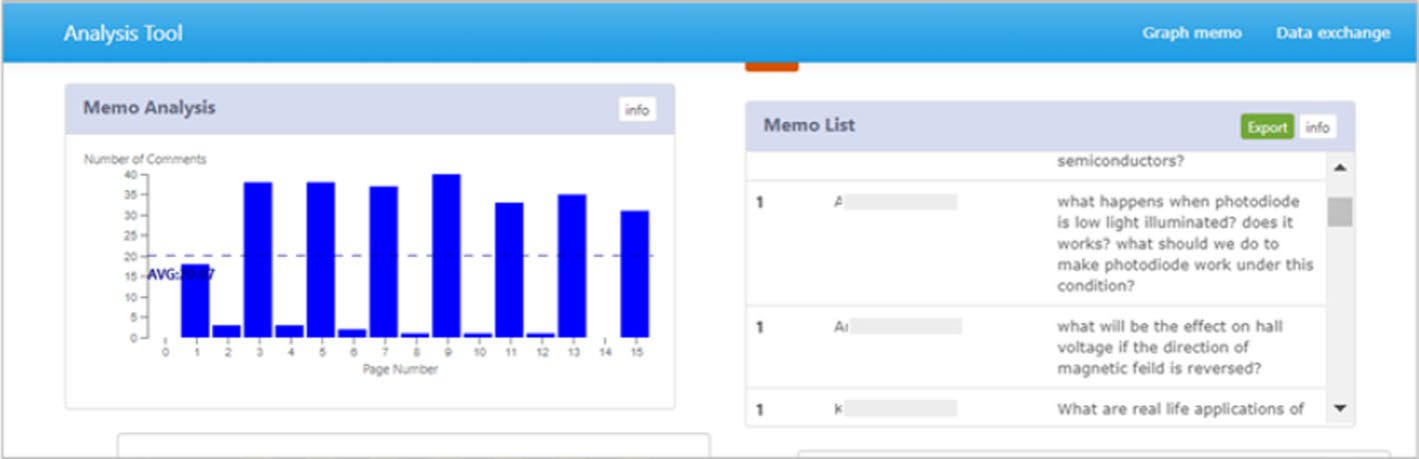
A screenshot of the LAViEW Dashboard section related to Memos

In the lecture focus phase, the instructor focused on students independently exploring the BookRoll features after her lecture session. As seen from Table 1, the full content discussion would happen over multiple lectures and the student activity would happen asynchronously between these activities. In the student activity, the students are being asked to reflect on the content and ask doubts or clarifications (see Fig. 3a). In our current design, the reflection spots of LeDs were implemented as “clarification spots” in BookRoll, to assist students to reflect or collate doubts using memo function. The doubts are then collected and analyzed using the LAViEW dashboard of the TEEL platform (see Fig. 4). Based on the analysis, the instructor provided feedback to the learners in the subsequent face-to-face (f2f) lecture session and thereby closed the learning loop.

### Problem-solving focus phase (P phase)

In the problem-solving focus phase, the instructor focused on a structured problem-solving activity using BookRoll memos and thereby facilitating student practice. Being a blended delivery mode, the entire content discussion is spread over multiple lecture sessions. However, a distinct difference between the clarification spot in the previous phase and “Reflection Spot” in the current phase is the nature of activity designed. The reflection spot activity requires students to solve a problem, which is similar to the problem discussed during the session, and upload the solution as a BookRoll memo. Thus, the activity allows them to perform micropractice. The LAViEW provides the instructor access to these solutions and she can then design the subsequent lecture session and close the learning loop. A generalized feedback on the student solutions were provided in subsequent f2f lecture sessions.

### LeD phase (L + P phase)

In the LeD phase, the instructor integrated both “clarification spot” and “reflection spot” within the same session. The analysis of learner memos in the clarification spot happens within the session, and the feedback is provided by the instructor based on the analysis. The creation of the memos by the student and subsequent analysis by the instructor happens asynchronously. The instructor then provides her feedback on the solutions in the subsequent session.

## Research methods

### Context

A single group study was conducted with purposive sampling of freshman undergraduate engineering (B.Tech.) students with the specialization (major) in electronics and communication at an autonomous institution in India. A total of 58 students were enrolled in an Engineering Physics course that was part of their second semester curriculum. Out of the 58 students, 83% were boys and the 17% were girls and they belong to the age-group ranging from 17 to 19 years. The course consisted of five modules, out of which first three and a half modules were taught during the pre-lockdown and the last one and a half module was taught during the lockdown. To address our RQs, we selected the target topics taught during three different teaching phases: lecture focus, problem-solving focus and the LeD focus across the semester. For performance score analysis, the learning test after each of the teaching methods was compared. The topic equivalence was checked along with the similarities in prerequisite knowledge for each topic verified with the following aspects: (1) learning time required, (2) complexity level, (3) similarity in prerequisites of knowledge to learn the topics. We also triangulated the student perception about the equivalence and difficulty levels for both the target topics chosen. Additionally, the format of the assessment (multiple choice questions—type) and the knowledge level of the post-tests conducted for each phase were kept equivalent.

### Data collection and analysis overview

The two major tools from the TEEL platform used were—BookRoll (eBook reader) and LAViEW (Analytics Dashboard). The goal of the pedagogic design was to achieve a sustained student engagement and learning, especially while abruptly transitioning to a fully online mode of learning during the lockdown. The BookRoll (eBook) activities generate learning logs of each student action and are used as a metric for student engagement on the platform. The MOODLE (LMS) provides instructors with the feature of online quizzes that will generate the learning data and are used as a metric for student performance on the learning content. To clearly distinguish the current study from our earlier results (Kannan et al., 2020a), we had performed a purposive sampling of the total population of learners (*N* = 58) and categorized them into high and low scorers based on the marks obtained in the two mid-semester summative assessment tests each conducted for a total of 30 marks. The contents of the assessment tests are the same as that was discussed in the course over a 3- to 4-week time period before the assessment. It required students to give detailed subjective answers, which was later evaluated by the course instructor. The students were then divided into high and low scorers based on the consolidated test scores of these assessments. If a student scored above the median score for the test, they were classified as high scorers, else they were low scorers.

To answer the RQ-1: “Is there any difference in engagement between low and high scoring students during the lecture focus, problem-solving focus and LeD focus phases?”, the following data were collected from the LAViEW analytics dashboard across each of the teaching phases: count of specific student log data in the BookRoll tool, i.e., the number of unique learners, unique learning content and time spent by the learners to manually visit the individual pages of the BookRoll materials provided. To answer RQ2: “What is the variation in the learning performance of low and high scoring students in assessments conducted at the end of each of the three phases—Lecture Focus, Problem Focus and LeD Focus?”, we look at the marks obtained by students in the quizzes administered at the end of the each phase using the MOODLE in the TEEL platform. These learning tests served as an indicator of the content knowledge acquired in individual topics. The test performance provided us with three strata of learners—learners who scored above the median (Strata 1), learners who scored below the median (Strata 2) and learners who were absent (Strata 3). We further explicate a transition pattern between these three strata of students across the three phases using the iSAT tool (Majumdar & Iyer, 2016). To answer the RQ-2, we also performed a qualitative analysis of learner artifacts (annotated as memos in BookRoll) generated during each of the teaching phases.

## Result and interpretation

The summative assessments were out of 15, and the median of this assessment was found to be 7.5. This resulted in 28 learners being identified as low performers and 30 learners being identified as high performers.

### Engagement analysis

**Fig. 5:**
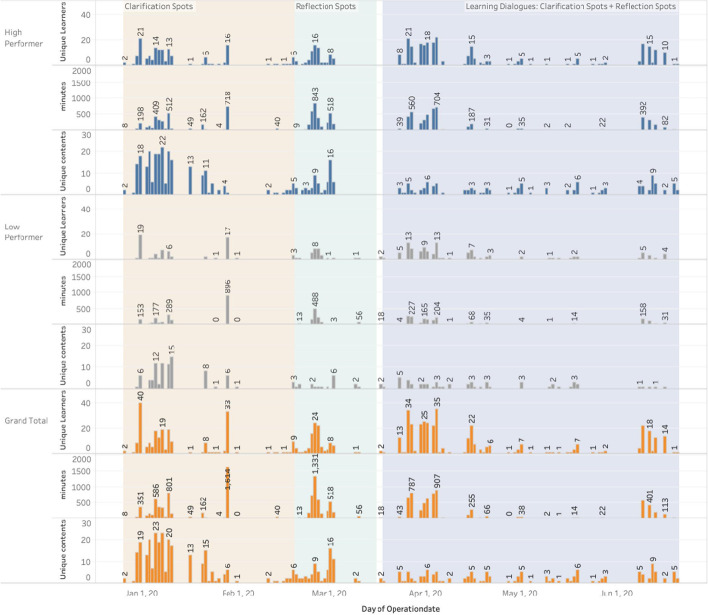
Student engagement across different teaching phases (L, P, L + P) as extracted from the eBook tool

A two-way analysis of variance was conducted on the influence of the type of different teaching phases (L, P, L + P) and learner’s prior performance levels (high and low) on their average engagement in terms of minutes spent (see Table 2) and the contents browsed on the online platform (see Table 3) by each learner. Results indicated significant effects of the different teaching phases on minutes spent (*F* = 8.46, *p* < 0.01, *η*^2^ = 0.111) and content browsed (*F* = 9.635, *p* < 0.01, *η*^2^ = 0.129). However, there was no significant difference found between the high and the low performing groups on either minute spent (*F* = 0.103, *p* = 0.311) or content browsed (*F* = 0.005, *p* = 0.942). Also, there were no interaction effects either for minute spent (*F* = 2.167, *p* = 0.119) or content browsed (*F* = 0.203, *p* = 0.817). Further post hoc analysis revealed the difference across the teaching phases. For time spent, problem-solving focus (*M* = 29.063 min, SE = 4.737) was significantly higher than lecture focus phase (*M* = 21.303 min, SE = 3.4) and LeD focus phase (*M* = 9.980 min, SE = 2.51). For content accessed, lecture focus phase (*M* = 2.34 contents, SE = 0.262) was significantly higher than problem-solving focus phase (*M* = 1.260 contents, SE = 0.334) and LeD focus phase (*M* = 0.925 contents, SE = 0.192). The detailed engagement variation for both high and low scorers over the period of time is shown in Fig. 5. Each teaching phase is highlighted in orange (L), green (P) and blue (L + P) background colors, respectively.

**Table 2:** Results of two-way ANOVA of engagement with teaching phase and performance level

Cases	Sum of squares	*df*	Mean square	*F*	*P*	*η*^2^
Teaching phase	7428.367	2	3714.183	8.460	< .001	0.111
Performance level	453.676	1	453.676	1.033	0.311	0.007
Teaching Phase*Performance level	1902.786	2	951.393	2.167	0.119	0.028

**Table 3:** Results of two-way ANOVA of average content access with teaching phase and performance level

Cases	Sum of squares	*df*	Mean square	*F*	*P*	*η*^2^
Teaching phase	49.307	2	24.654	9.635	< .001	0.129
Performance level	0.014	1	0.014	0.005	0.942	0
Teaching Phase*Performance level	1.038	2	0.519	0.203	0.817	0.003

We suspect that the familiarity gained by the students with the learning design and the tools from the L and P phase activities would have had a great bearing on this result. The students were found to participate in most of the asynchronous activities, as the instructor could provide feedback on their learning using the analytics dashboard of TEEL platform. Our results pointed out that the students made use of technology-supported learning activities more effectively during the L + P phase, despite being in the state of transition from the blended (before lockdown) to fully online mode of teaching (during lockdown).

### Performance analysis

#### Learning test scores at the end of the topic

**Fig. 6:**
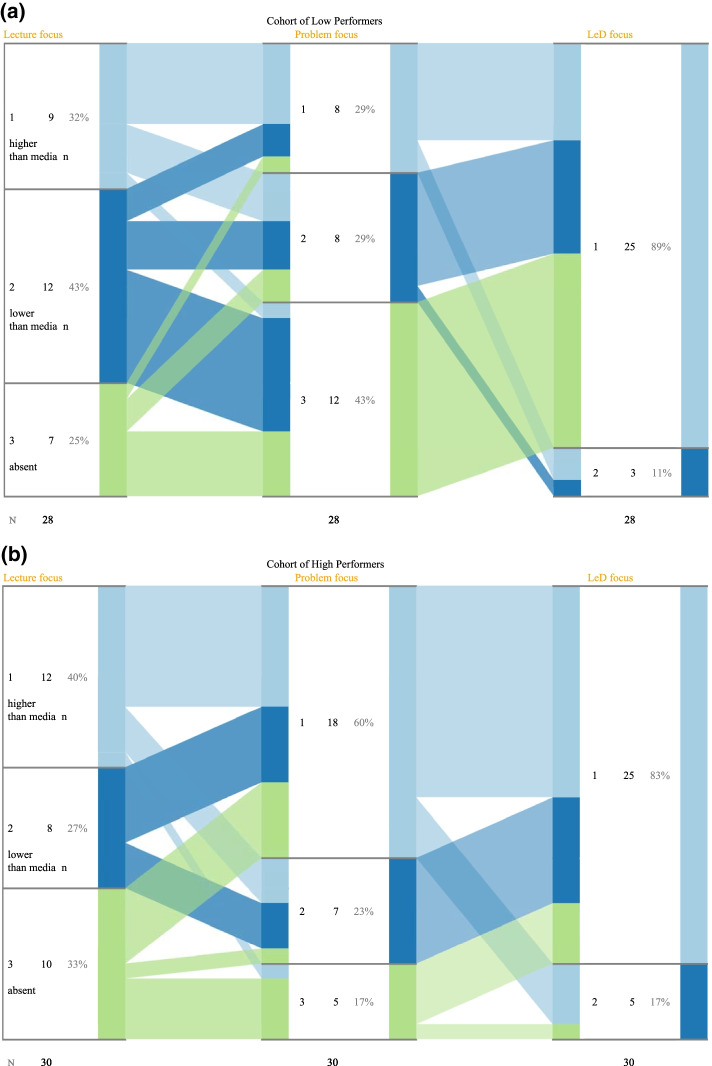
iSAT analysis of test scores for post BookRoll activity after the lecture focus, problem focus and LeD (L + P) focus phases for** a** transition pattern of the low performers.** b** transition pattern of the high performers

The iSAT transitions were identified for both high and low scorers (Fig. 6). Figure 6a shows the transition pattern between L, P and L + P phases for low performers. Out of the total number of low performers, *N* = 28 students, 9 (32%), 12 (43%) and 7 (25%) fall into the strata 1 and 2 and 3, respectively, in the initial L phase.

However, it is observed that the overall percentage of strata 1 first dipped to 29% and then increased to 89% during the P and L + P phases, respectively. Out of the 25 (89%) in the L + P phase, nearly 6 students were already in strata 1 in P phase while 7 students (25%) showed an upward transition from strata 2 of the P phase. Further, almost all the 43% of strata 3 transitioned to strata 1 during the L + P phase. Figure 6b shows the transition pattern between the L, P and L + P phases of high performers. Out of the total number of high performers, *N* = 30 students, in the initial L phase 12 (40%), 8 (27%) and 10 (33%) fall into 1 and 2 & 3 stratas, respectively. There is an increase in strata 1 percentages to 60% and 83% in P and L + P phase, respectively. In addition, nearly 42% in strata 2 showed an upward transition from L to P phase and 23% of learners in strata 2 showed an upward transition to strata 1 during the L + P phase. It is to be noted that in strata 3, 43 (% absentees) transitioned into strata 1 in the P phase.

To further verify statistically whether this is a promising trend, we then compare the learning test scores (conducted as quizzes in MOODLE) of a subset of students (*N* = 31) whose scores were present for all the three L, P and L + P phases. The mean scores across the three phases showed differences—Mean = 6.43 out of 10, SD = 1.58, was obtained for L phase; Mean = 7.47 out of 10, SD = 1.17, was obtained for the P phase; Mean = 9.51 out of 10, SD = 1.73, was obtained for the L + P phase. The statistical analysis using the one-way ANOVA of the test scores in all the three phases was carried out. The results showed that the differences were statistically significant (See Table 4).

**Table 4:** Statistical difference analysis of the test conducted after the L, P and L + P teaching phases (*N* = 31)

Activity	Mean (out of 10)	SD	*V*	SS	*df*	MS	*F*	*P* value*	F-crit
Lecture focus (L)	6.43	1.58	2.645						
Problem focus (P)	7.47	1.17	1.453	16.66		16.66	8.12	0.006	4.00
LeD (L + P)	9.51	1.73	3.195	64.740	1	64.74	27.85	0.00	4.00

^*^Significant at *p* value < 0.05

#### Analysis of student memos in different activities

In our instructional design, memo-based BookRoll activities were designed and implemented within the TEEL platform (LMS). The seed knowledge was primarily provided as the learning material in BookRoll, which was then followed by the teaching sessions delivered via blended or online mode. The students were instructed to create clarification questions or artifacts as the BookRoll memos (at the “clarification spots” activity) explicitly around the seed knowledge being provided during the teaching sessions. The analysis of generated text artifacts is very common in discourse analysis and problem posing research literature. The discussion forum analysis literature suggests techniques like domain ontology and text mining (Li et al., 2009; Breno et al., 2011), while the problem posing literature suggests categorization schemes to classify the type of question being asked (Graesser et al., 2008; Mishra & Sridhar, 2015).

In the current work, we adapt a similar approach as proposed by Mishra & Sridhar, 2015, to qualitatively analyze the student memos based on the quality or type of knowledge being requested. We examined the different terms formulated within the student memos to qualitatively check if the students could develop an ability to construct an additional knowledge related to the seed knowledge. We then collected all the memos across different BookRoll activities across each teaching phase. We removed the redundant or irrelevant memos from the total collection of memos and then performed an inductive approach to analyze them between different performing groups. A representative example of a few selected memos generated by both high (Student 1 and 3) and low scorers (Student 2) are shown in Table 5.

**Table 5:** Qualitative analysis of memos posted by students

Phase	Example of memos of learners
Lecture focus (L)	Student 1—“what is exact meaning of flux?; How to derive Coulomb’s law from Gauss law”; “Why is there change in magnetic flux” “What are disadvantages of Coulombs law”; How Coulomb’s law equation equal to gravitational equation ?” Student 2—“How is the flux zero in zero position”; “Why is the potential difference between the two charges is independent from the path h taken?”; “Why is the gradient operator not a vector in itself?”
Problem focus (P)	Problem Statement: Consider a new oxide dielectric material having an electric permittivity value of 1.74 × 10^–10^ C^2^/N-m^2^. Determine its dielectric constant and electric susceptibility Student 1 Solution: Dielectric constant, *k* = 19.66 susceptibility = 165.141 * 10^−12^ Student 2 Solution: 58.05 * 10^−12^ c^2^/n-m^2^
LeD focus—L	Student 3 – why does the recombination of an electron and a hole generate light in direct band gap semiconductors and generate heat in indirect band gap semiconductors?
LeD focus—P	Student 2—“What will be the effect on Hall voltage if the direction of magnetic field is reversed?” “what happens when photodiode is low light illuminated?. does it works? What should we do to make photodiode work under this condition?” “How does charge accumulation balance Lorentz force?”; “How can we tell how sensitive a photodiode is?” Reflection Spot (Conceptual): Why are direct band gap semiconductors preferred to make light emitting diodes (LEDs)? Student 3—“In direct semiconductors the momentum vector k is aligned along the CB and VB which would make it easy for the LED to pass as much as current into it.” Student 2—LEDs are mostly made from direct semiconductors because no change in momentum is required for an electron in the conduction band to recombine with a hole in the valence band Reflection Spot (Problem): When an electric field of 160 V/m is applied to a semiconductor sample whole type is unknown. The sample exhibits a Hall coefficient of value—0.0125 m^3^/C (i) Whether the semiconductor is N-type or P-type? (ii) Determine the current density in the sample, assuming the mobility of electrons (*μ*_e_ = 0.6 m^2^/V s Student 3 solution—(i) “As the hall coefficient and the hall voltage is negative the semiconductor is N-type” (ii) *J* = − 7680 A/m^2^ Student 2 solution—“Charge carrier concentration (*n*) = 2.0862 * 10^25^ carriers/m^3^ ii. drift voltage (Vd) = 0.12 m/s; hall voltage (Vh) = 2.66 * 10^−6^ V or 2.66 microvolts.”

#### Memos in lecture focus (L phase)

In the L phase, most of the students created clarify-type questions in the BookRoll memos (see Table 5 for examples). Even though there were a good number of clarification memos, most of them prefixed the question text with “what, why or how” with the content provided in BookRoll (for instance, “What” is exact meaning of flux, for a topic “electric flux”). There was no evidence of students constructing their own new knowledge which was an expected outcome of this activity. It was also observed that, similar to the engagement results, the total number of memos created by the low scorers were less in comparison to high scorers in this phase. A possible reason for such a behavior could be that the students were mostly exploring the technology (BookRoll or the MOODLE) tools provided to them rather than keeping their focus on learning contents deeply.

#### Memos in problem focus (P phase)

In the P phase, we had provided a set of model problems for concept acquisition during the lecture session which was subsequently followed by the similar problems provided as an assignment (termed as “Reflection Spot” activity). The students were instructed to learn the concepts from the model problems first before finding solutions to these assignment problems. The students need to enter the end solutions of reflection spot questions using the memo function of BookRoll. Further, they were asked to justify their own solutions. The number of students who provided memos in the reflection spot activities increased considerably, and we found that there were more correct answers with highly relevant explanations (see Table 5).

#### Memos in LeD focus (L + P phase)

Analysis of artifacts generated during the LeD focus phase showed that the students were able to generate memos with better thinking and questioning skills. For example, students generated memos like “Why the lifetime of charge carriers in the direct band gap semiconductor is less than indirect band gap semiconductor,” where they compared two different related seed concepts being taught to them. In another instance, two other memos are shown where students posed the following questions—“what will be the effect on hall voltage if the direction of the magnetic field is reversed?” and “What happens when the photodiode is low illuminated? Does it work? What should we do to make photodiode work under this condition.” In these memos, the students had applied the seed concepts to modify or vary the given conditions (magnetic field in Hall effect or low illumination effects on photodiode) that were discussed in the BookRoll content. Analysis of all these memos clearly revealed that the clarifications raised by students were more focused, exploratory-type and relevant to the session contents as compared to the previous L phase. It also showed that the students had paid attention to the preceding seed knowledge being taught and had put sufficient thinking on contents before raising the doubt. In fact, there were also memos pointing to concepts that were going to be covered in the next teaching sessions. In this phase, the clarification spot was addressed instantly by the instructor during the synchronous online session, which also helped students to better focus on the subsequent topics dealt in the same session.

### Instructor reflection

“A teacher’s reflection on their practice would offer a powerful lens for understanding in-the-moment decision making as well as decisions that are made by reflecting back on past action or planning for future action” (Kopcha et al., 2020). In the current study, we have primarily looked at the evolution of the strategy and its impact on learning and engagement. With the course instructor also being the lead author of the paper, we now present a first person account of the learning from the entire implementation.

The use of the BookRoll eBook Reader along with the LAViEW Analytics dashboard as an integrated tool in the TEEL platform has greatly facilitated the orchestration of our pedagogical design. The proposed LeD with BookRoll design has engaged the students in both practice and reflection without worrying about access credentials to multiple learning tools. Thus a key focus while moving to ERT should be to find such integrated environments first and then focus on active learning pedagogical designs that will improve the learning outcomes. While implementing the technology-enhanced learning design, it is important to adapt a few scaffolding steps specific to the L, P and LeD focus phase. For example, during the L focus phase, the scaffolding was done to get the students more comfortable with the tools and pedagogy being adapted. The first few icebreaking activities using the tool (BookRoll in this case) were designed that are more flexible and fun-based. These activities need not focus on student’s learning the content, rather it can focus on getting student’s familiarized with pedagogical design and exploring the features available in the technology tool. These sessions should provide clear instruction or step-by-step guide to students on how to use various technology features (memos, bookmarks, highlights, etc. in BookRoll). Provide demonstrations on how the LAViEW Analysis dashboard of TEEL platform could be used to extract the student artifacts and to provide feedback of learning. More guidance is required to students across the initial tasks to help them troubleshoot any technical issues and to encourage an active participation, as it is crucial for students to recognize the intended usage of any tool and how to use it best to help in their own learning tasks. Scaffoldings during the P focus phase were mainly focused to attain better student’s engagement in the BookRoll activities. The problem-solving BookRoll activities were designed as targeted assignments that were aligned to the conceptual learning. To ensure an effective engagement, the students were further instructed with a mandatory submission of the elaborate solutions of assignment problems in the MOODLE course page, which were subsequently evaluated and the grades of the same were included as continuous assessment marks. The scaffolding during the LeD focus (L + P) phase was required to meet the challenge of sustained student engagement in the synchronous online teaching sessions. In the current course, common misconceptions from the student annotations were available in the LAViEW dashboard and this helped in providing constructive feedback to the students during the following online teaching sessions. While the LeD focus activities were implemented, instructor shared the student memos gathered in the LAViEW dashboard during the synchronous online sessions to encourage the low-engaging students to reflect on their own or peer’s memo. During these sharing sessions, the instructor realized the importance of repeating these in a step-by-step fashion across a few more topics, so that the students were clear with the pedagogical design as well. The teacher could use different indicators for engagement from the LAViEW dashboard, for example, the reading time, number of events, long events, memos created in BookRoll, all of which revealed that the scaffolding strategies had greatly influenced the student’s level of participation.

Reflecting on the entire process at the end of the semester, technology integration is perceived as a really complex process. With technology becoming a mediator between instructors and students, even a simple strategy can feel like a complex and difficult task to accomplish. Unlike a face-to-face classroom, it is difficult to initially get a sense of learner engagement in the strategy and hence there is a disconnect. The instructor started with designing a strategy that is simple and easily understandable by both teacher and the students, continuously refine them by checking the engagement indicators at the end of each class in LAViEW, to get a better sense of the class participation. This helped to put together the teaching–learning strategy (LeD focus phase) while we moved to complete online teaching during the lockdown.

## Discussion and conclusion

The rapid transition to emergency remote teaching had an impact on both learners and teachers. However, from our current study we see that persevering with a technology and further working to evolve pedagogies around the technology features permit instructors to make the optimum use of technology-enhanced learning during ERT. Previous study reported analysis of TEEL platform logs collected from a Japanese public university (Majumdar et al., 2021) as well as public junior high school (Kuromiya et al., 2022) contexts during the ERT period. The study at the university level highlighted engagement of learners in different domains of subject with learning design utilizing various features of BookRoll. It had recommendations regarding inclusion of audio and in-reading activity exercises for higher engagement in different subject domains while conducting synchronous or asynchronous sessions. However, the study could not confirm specific pedagogy of more than the 240 courses whose data was analyzed. The study at the junior high school level analyzed engagement from learning logs and perception of students from three grades. It recommended future implementation strategies of remote learning with ebook materials which is more focused in the educational context of Japanese junior-high schools. In this paper, we look at the evolution of teaching–learning practices around the TEEL platform by an instructor to encourage active learning among her students. We specifically look at how high and low performers in the course (as per two summative tests taken during the course) performed in an end of phase learning test on the topics which had specific pedagogical strategies.

### Summary of findings

The analysis of engagement over the three phases of the course through a two-way ANOVA shows that the teaching phase had a significant effect on content access and engagement duration, while the scoring levels of students did not have a direct effect. As seen from the design of activities and data collected:

- Learners were provided with more content initially due to which the number of content accessed was high in the L phase (*M* = 2.34 contents, SE = 0.262). However, average time spent on content was less here (*M* = 21.303 min, SE = 3.4).
- Learners were provided with problem-solving activities in P phase, which was seen to create higher engagement (*M* = 29.063 min, SE = 4.737) even though the content access was slightly lower (*M* = 1.260 contents, SE = 0.334).
- Learners were provided with both content and problem-solving activities in LeD focus phase, which had lower engagement (*M* = 9.980 min, SE = 2.51) and content access (*M* = 0.925 contents, SE = 0.192) as compared to L and P phase. The lower rates were expected as the knowledge transmission and activity happened synchronously during the online classes done during the ERT phase.

The learning performance transitions also showed that a significant proportion of learners moved from low to high marks strata (at the end of topic learning test) after the L + P phase. The series change was more steady in the high-scorer category with the transition percentages being 40%, 60% and 83% for the L, P and L + P phase. The mean scores of the learning test also showed statistically significant improvement from mean value of 6.43 for L phase to 7.47 in P phase to 9.51 in L + P phase (all out of 10). One-way ANOVA test on the mean scores confirmed that these differences are statistically significant.

Learning and engagement are two important metrics that are consistently examined by researchers while looking into effectiveness of technology-enabled interventions (Anderson, 2009; Whipp & Lorentz, 2009; Lin et al., 2017). The results in our current study on learning and engagement show that as the strategy evolved over the semester there is an increase in both these metrics. There is also qualitative evidence, from the BookRoll memos, that indicates that the nature of clarifications posed by the student as memos varied from surface-level questions (in L phase) to a more focused, exploratory and highly relevant content (in L + P phase). As the strategy evolved over a semester, the experience with both technology features and pedagogic strategies has exposed the learners to the idea of self-reflection which has a positive impact on the learning outcomes (Means et al., 2009).

The quantitative results on engagement and learning are useful even in such a situation as this acts as an indicator of effectiveness of the learning design in an online setting.

Various researchers have recently reported on evaluating the student engagement and learning in the context of university-level educational courses during COVID-19 emergency remote teaching period. These studies have focused on how students self-regulated learning capabilities (Zhang et al., 2021), self-efficacy (Lazarus, 2021), online technology platforms (Long et al., 2021) impacted the students' engagement and learning. However, we find that our results on engagement and learning could be compared with the findings reported by (Long et al., 2021). Their results offer insights that a positive relationship between the student reflection and interaction within an online learning technology is a key component to significantly promote the self-learning experience. Similarly, it has been reported by Baber (2021) that there is a positive correlation between the interactive learning activities with the learner’s satisfaction which plays a significant role in determining the student’s learning outcomes in an online environment. On the contrary, a few studies found that learner–learner or learner–instructor interactions have no significant effect on learner’s satisfaction and engagement on different open online educational courses (Kuo et al., 2014; Gameel, 2017).

### Implications and perspectives for the future

The following are the major implications that we can share with the larger practitioner community:

- Identifying technology platforms or learning environments that integrate learning analytics explicitly is crucial for making real-time improvements in pedagogy.
- Instructors should focus on incremental (or step-by-step) improvement in online pedagogical designs as learners have to get adjusted to new pedagogy and technology.
- The pedagogical design should have explicit activities for both practice and reflection on the learning content. Students should first be exposed to practice activities and once they are familiarized with the technology, then instructors should focus on reflection.
- Instructors should try to elicit misconceptions from students during both practice and reflection activities
- Direct instructor feedback should be incorporated wherever possible to address the misconception. The feedback can happen either as an overall summary session or as personalized individual feedback.

**Fig. 7:**
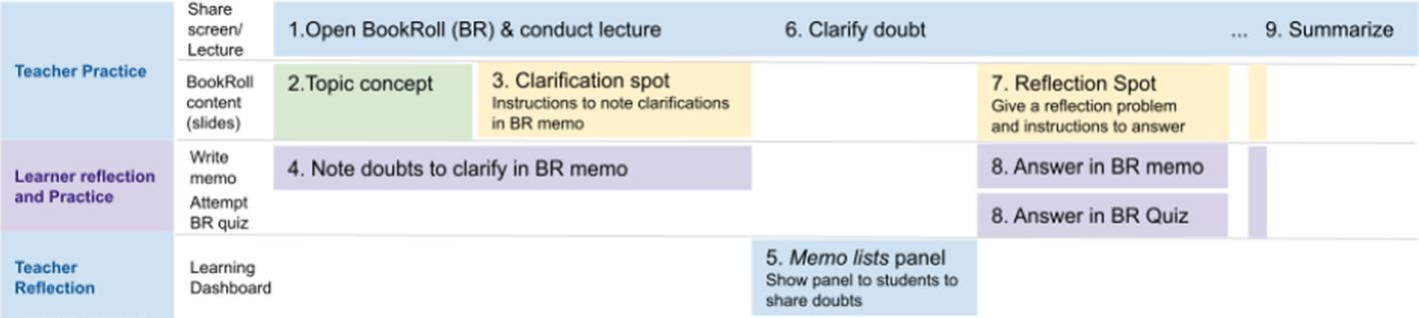
Integrated view of LeD focus phase orchestrated in the TEEL platform

Figure 7 shows an integrated view of the pedagogic adaptation done by the instructor while orchestrating LeD through BookRoll across the semester. In L phase (steps 1–6), the students were asked to explore the BookRoll and create memos that resulted in less engagement among learners even though there were sufficient cues (Clarification Spot as given in step 3). The pedagogy that the teacher implemented during this phase attempted to directly generate learner reflection at the clarification spots (as BookRoll memos). The analysis of the artifacts (from LAViEW dashboard) shows that learners did not do deep reflection rather stayed at the surface, often copying from the same content.

This necessitated a shift in pedagogy toward explicit practice that forced students to work on problems similar to the ones discussed in class and thereby promoted active learning. Thus, in the P phase (steps 1, 7–9), the focus was shifted to permitting student practice that allowed students to work with memos more (Reflection Spot in step 7). This enabled the instructor to expose learners to multiple opportunities of practice and feedback with the BookRoll tool.

Thus, in the L + P phase (steps 1–9), the instructor carefully integrated the pedagogies in the L and P phases and ensured that learners obtained a consistent experience. This familiarity and consistency in experience is crucial while adapting the teaching strategy to technology affordances so that students find it more appealing (Ertmer, 2005). Learners who were already familiar with both the pedagogy and technology, now engaged more freely during the L + P phase. The increased access of the platform and larger number of memos created in this phase are evidence to this. Thus, when the instruction was shifted to ERT, students had been sufficiently exposed to technology-enabled active learning strategies in the TEEL platform and were able to continue without many issues related to technology unfamiliarity (Vera et al., 2020). This helped in generation of more meaningful memos and better engagement practices from the learners.

In Fig. 7, we also see that the instructor has provided opportunities for reflection and practice both for learners as well as herself. The provisioning for teacher reflection is critical for them to make better informed decisions on their practice (Schőn, 1987). As seen from the instructor’s reflection, the integration of the LAViEW dashboard permitted the instructor to do immediate analysis of the student responses, provide feedback in the subsequent lectures and also improvise on the existing strategy. The careful integration in the L + P phase was possible primarily because the instructor continuously reflected on her prior practice and was exposed to the technology for a significant amount of time prior to this. It is also significant to note that the entire online pedagogy had evolved from smaller chunks of initial practice that was well knit through the learnings of the instructor during the implementation in the initial phases. Thus, the need for both technology and pedagogy that supports reflection is all the more critical in an online setting as there is a growing sense of discomfort among both learners and teachers due to the sudden shift (Vera et al., 2020).

### Limitations

The study progressed from a blended environment to a fully online environment (due to COVID constraints). This limits any quasi-experimental design to compare strategies across phases as there are too many confounding factors. Hence, we have taken the approach of showing the evolution of the strategy rather than comparing them.

Our current quasi-experimental single group study is limited to the purposive sampling. Even though the overall student strength was high, not all students had participated in the post-test conducted during the lockdown. This made random sampling difficult. As the strategy evolved over the semester, with an abrupt transition due to lockdown, a pre–post-research design was not possible. In our future implementations, we will plan for two-group pre–post-design and measure the learning gains across each phase to refine our results.

## Conclusions

Research in online learning has already provided us with sufficient pointers on how to make the teaching–learning process more effective and enjoyable for the learner. The sudden transition to “Emergency Remote Teaching” had left both instructors and students with a sense of confusion, with many ERT practices trying to mimic the face-to-face classroom (Hodges et al., 2020). The evolution of the pedagogy of LeD orchestrated with the eBook reader provides us with a significant lesson on the need for promoting both learner and teacher reflection in our teaching–learning practices with technology. With the teaching–learning expected to continue in an online mode for a longer period of time, the institutions should start exploring integrated technology solutions, like the TEEL platform, that will have features to promote such reflective practices.

## Data Availability

Not applicable.
